# An Adaptive and Time-Efficient ECG R-Peak Detection Algorithm

**DOI:** 10.1155/2017/5980541

**Published:** 2017-09-06

**Authors:** Qin Qin, Jianqing Li, Yinggao Yue, Chengyu Liu

**Affiliations:** School of Instrument Science and Engineering, Southeast University, Nanjing 210018, China

## Abstract

R-peak detection is crucial in electrocardiogram (ECG) signal analysis. This study proposed an adaptive and time-efficient R-peak detection algorithm for ECG processing. First, wavelet multiresolution analysis was applied to enhance the ECG signal representation. Then, ECG was mirrored to convert large negative R-peaks to positive ones. After that, local maximums were calculated by the first-order forward differential approach and were truncated by the amplitude and time interval thresholds to locate the R-peaks. The algorithm performances, including detection accuracy and time consumption, were tested on the MIT-BIH arrhythmia database and the QT database. Experimental results showed that the proposed algorithm achieved mean sensitivity of 99.39%, positive predictivity of 99.49%, and accuracy of 98.89% on the MIT-BIH arrhythmia database and 99.83%, 99.90%, and 99.73%, respectively, on the QT database. By processing one ECG record, the mean time consumptions were 0.872 s and 0.763 s for the MIT-BIH arrhythmia database and QT database, respectively, yielding 30.6% and 32.9% of time reduction compared to the traditional Pan-Tompkins method.

## 1. Introduction

Electrocardiogram (ECG) can describe the electrical activity of the heart and is an essential tool for the diagnosis of cardiovascular diseases (CAD). With the rapid development of wearable and wireless ECG techniques, real-time and routine ECG monitoring is attracting more and more attention due to the increasing popularization of medical health, especially for the elderly people [1]. Recent years, lots of publications about ambulatory ECG monitoring devices have been reported [2–4], aiming to automatically monitor the heart activities and give the feedback of any CAD early warning in real time. However, this application still needs significant development due to the challenge of unexpected noise effects in ECG signal, such as baseline drift, electrode motion and stretching, motion artifacts, and muscle noise [5, 6], which impedes the automatic ECG processing technology to perform effectively. The primary sources of noises are electrical activities of muscles and baseline drift caused by respiration, poor contact of electrodes, and equipment or electronic devices [7, 8]. Electrode movement alters the signal baseline and brings the signal fluctuate perpendicularly to the baseline. If the electrode moves drastically enough to drop from the skin, baseline drift will overwhelm the signal and waveform distortion occurs [9]. Motion artifact is generally attributed to the variation of electrode-skin impedance during a subject's motion. Changed impedance will be treated by the ECG amplifier as a different input, resulting in impedance mismatching and difficult identification of irregular fluctuation on small amplitude waveforms, such as P wave and T wave [10, 11]. Consequently, noise removal is the preliminary issue to consider for in ECG signal processing.

ECG features are essential characteristics for CAD diagnosis. R-peak detection is the datum since all other features are extracted after the R-peak location [12]. Accurate R-peak detection is critical for arrhythmia diagnosis such as atrial premature contraction, tachycardia, and bradycardia [13]. Nevertheless, efficient R-peak extraction is still difficult in the dynamic and noisy environment due to the time-varying waveform morphology. This would be more difficult when ECG signal is overwhelmed by noises with similar frequency in energy distribution [9].

Over the last decades, numerous techniques have been proposed for R-peak detection. In [14], a thorough review on R-peak detection methodologies for portable, wearable, battery-operated, and wireless ECG devices was elaborated. The authors claimed that the thresholding methods were regarded as the most computationally efficient. However, the suitable parameter settings for thresholds were difficult. The most widely used R-peak detection method, proposed by Pan and Tompkins [15], is the Pan-Tomkins method. It is a threshold-based method with low complexity. Other algorithms of R-peak detection can be classified as pattern recognition [16, 17], wavelet transform [18], mathematical morphology [19], and digital filter [20]. In [10], a real-time R-peak detector using adaptive thresholding was proposed. This algorithm consisted of preprocessing to initialize R-peak threshold and thresholding to adaptively modify the threshold. It achieved sensitivity and positive predictivity higher than 99.3%. In [21], a different interference-based method was developed. This method could effectively distinguish R-peaks from high amplitude noises but failed to detect R-peaks when abrupt jump of baseline appeared. Some researchers also conducted ECG feature extraction without predenoising [22, 23]. The detection accuracy could reach up to 94.8%, although much lower than that acquired from the denoised signals.

Time cost is important due to the fast-responding requirement in CAD early warning applications [4], especially in the real-time monitoring. Many ambulatory ECG devices are generally limited in power supply and computation [1]. The conventional feature extraction algorithms are, from a computational perspective, very intensive tasks, which are typically executed in mainframe-type computational facilities. A significant power expenditure component in such systems is the energy required by the radio front-end for supporting continuous data transmission, which may not allow a long-term sustainable operation. To this end, some researchers have attempted to develop algorithms of low computational load. Apart from the aforementioned methods, in [24], the authors presented a low-complexity ECG feature extraction approach for mobile healthcare applications. This technique was based on the combination of wavelet analysis and time-domain morphology principles. Except for high accuracy and precision, low computation and fast response are also needed in ECG feature extraction.

In this study, an adaptive and time-efficient ECG R-peak detection algorithm is proposed. The method takes advantage of wavelet-based multiresolution analysis (WMRA) and adaptive thresholding. WMRA is applied to strengthen ECG signal representation by extracting ECG frequency interval of interest from wide-range frequencies, which contain interference such as baseline drift and motion artifacts. All the noises produce considerable influence on the following thresholding operation. The adaptive thresholding is designed to exclude false R-peaks in the reconstructed signal by WMRA. The proposed algorithm was tested by the MIT-BIH arrhythmia database (MITDB) and the QT database (QTDB) [25]. Both accuracy and time consumption of the algorithm were evaluated. By exploring the time-frequency property of ECG, this study aims to conduct preliminary and tentative research on adaptive and time-efficient R-peak detection for low-quality ECG signals, promoting automatic ECG processing technology for clinical and daily use.

The remainder of the paper is organized as follows.  Section 2 elaborated the detailed procedures of the proposed R-peak detection algorithm. In Section 3, experiment setups were introduced, including the datasets and the evaluation indices.  Section 4 demonstrated the experimental results over R-peak detection accuracy, time consumption and time complexity, and the selection of optimal threshold coefficients.  Section 5 discussed the advantages and the potential limitation of our algorithm. The summarization of this study was presented in Section 6.

## 2. Proposed R-Peak Detection Algorithm

**Figure 1:**
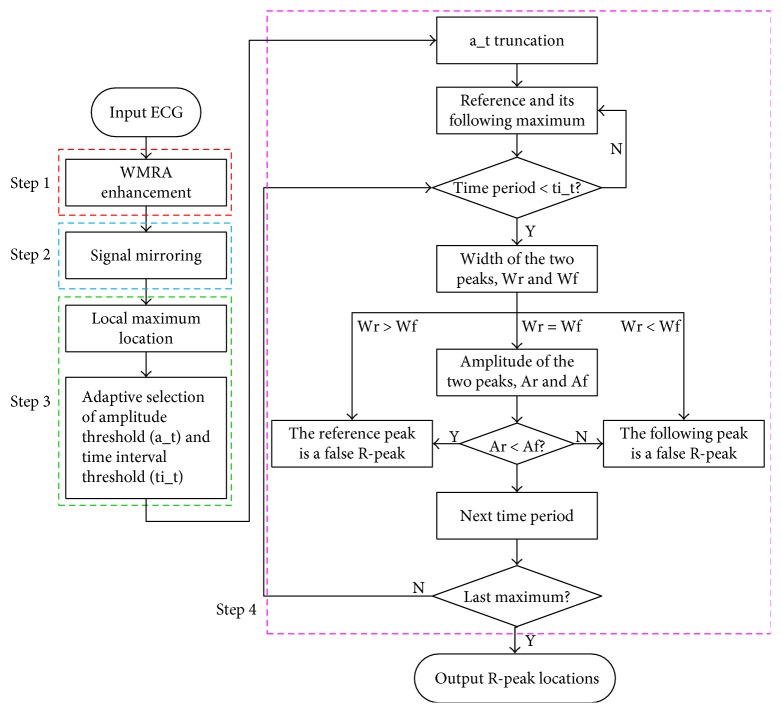
Block diagram of the proposed R-peak detection algorithm.

The R-peak detection system is described in Figure 1. The purpose of this study is to develop an algorithm which can effectively identify R-peaks mixed in different noises.

### 2.1. Step 1: WMRA Enhancement

WMRA enhances signals using wavelet transform to extract both time and frequency domain information. This method is very suitable for ECG processing since ECG is essentially nonstationary with small amplitude (0.01~5 mV) and low frequency (0.05~100 Hz) [26]. This method also provides low computational cost [27]. By WMRA, signal below 0.05 Hz and above 100 Hz can be filtered from the raw signal. These intervals are not the ECG frequency bands and contain most types of noises [28]. In addition, according to the Nyquist criterion, subfrequency band presented by each decomposition level is directly related to the sampling frequency *f*_s_ [26]. Consequently, the ECG signals, sampled at 360 Hz in MITDB and 250 Hz in QTDB as illustrated in [25], are all decomposed up to 8 levels in this study.

**Figure 2:**
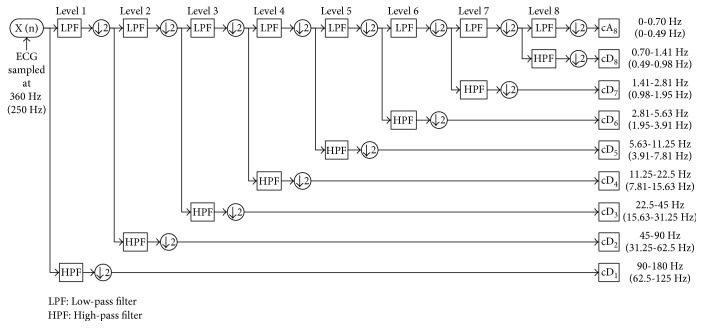
Decomposition process of the eight-level WMRA. The sampling frequency is decomposed into two subbands: high frequency of detail coefficient (cD_*j*_) and low frequency of approximation coefficient (cA_*j*_), both in Level *j*.

Figure 2 shows the decomposition procedure of eight-level WMRA by using *bior6.8* wavelet. For MITDB, cD_2_ ~ cD_8_ consist of frequency components in a range of 0.70–90 Hz, which is the ECG frequency band of interest. cD_1_ with frequency band 90~180 Hz and cA_8_ with frequency band 0~0.70 Hz are beyond the ECG frequency; they are not the considered coefficients containing baseline drift and other interference. Consequently, cD_1_ and cA_8_ are set to zeroes; cD_2_ ~ cD_8_ are kept for reconstruction. Similarly, for QTDB, cA_8_ with frequency band 0~0.49 Hz is set to zero; cD_1_ ~ cD_8_ with frequency components in a range of 0.49–125 Hz are kept. All the retained coefficients are then filtered by the wavelet shrinking threshold algorithm [29]. In this study, soft thresholding is adopted due to its good continuity and no Gibbs phenomenon on step points [30].

### 2.2. Step 2: Signal Mirroring

**Figure 3:**
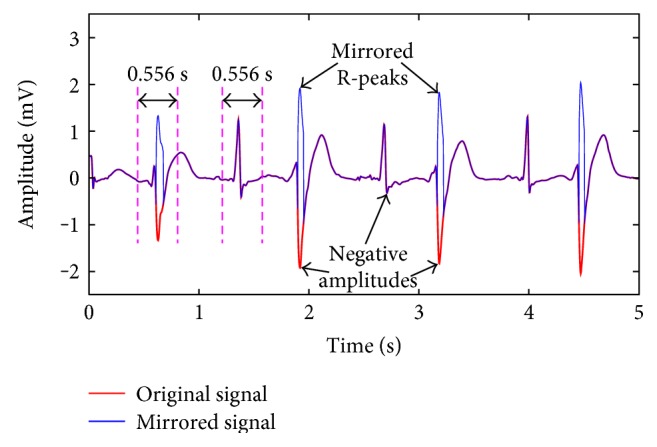
Signal mirroring example with comparison range of 0.556 s and amplitude multiple of 1.5.

For some ECG patterns, such as premature ventricular contraction (PVC) beat, R-peaks are presented with amplitude below the baseline but other features are above the baseline. To avoid the potential missing detection, signal mirroring is designed. The mirroring procedure for a PVC segment is described in Figure 3. Large negative amplitudes are mirrored by taking the baseline as their symmetrical axis. However, not all the negative amplitudes are mirrored, they should be significantly distinctive from adjacent negative values. This assumption is based on the fact that R-peaks have steep slopes while other waves such as P wave and T wave have gentle ones [10]. Steep slope means drastic increment and decrement on both sides of local maximum, and the slope is finished within several sampling points. If the absolute amplitude of a negative point is 1.5 times larger than that of the adjacent points within 0.278 seconds (0.278*f*_s_ points) before and after it, then the negative point will be mirrored.

(1) $$\begin{array}{c} Negative\left\{\begin{array}{cc} mirrored & if\forall \max\left\{0,k-0.278f_{s}\right\}\leq i\leq \min\left\{L,k+0.278f_{s}\right\},i\neq k,\exists \left|A_{N}\left[k\right]\right|\geq 1.5\left|A_{A}\left[i\right]\right|\\ unchanged & else \end{array}\right., \end{array}$$

where *L* is the signal length, *A*_*N*_[*k*] is the amplitude of large negative point with position number *k* in signal, 0 < *k* ≤ *L*, and *A*_*A*_[*i*] is the amplitude of point within 0.278 s before and after the large negative point.

In some literatures [15, 21, 31–33], authors recommend that signal with baseline drift removed could be squared to highlight the difference between true R-peaks and false ones, such as high-amplitude noise and high-amplitude P waves. However, this operation may not be suitable in our method due to the differences among R-peak amplitudes. If the signal is squared, amplitude values below 1 will be smaller than the original, and values above 1 will be enlarged. This increases the difference among true R-peaks and is adverse for the amplitude threshold to detect potential R-peaks, especially when a signal segment is mixed with large and small amplitude R-peaks.

### 2.3. Step 3: Local Maximum Location and Adaptive Threshold Selection

Local maximums are located by implementing first-order forward differential in the mirrored signal. The procedure is illustrated as follows.

(1) First-order forward difference is implemented on ECG signal with ΔECG[*n*] = ECG[*n* + 1] − ECG[*n*].
(2) For all the elements in ΔECG[*n*], values less than, equal, and more than 0 are replaced by −1, 0, and 1, respectively,
  (2) $$\begin{array}{c} \Delta ECG\left[n\right]\leftarrow \mathrm{sgn}\left(\Delta ECG\left[n\right]\right)=\left\{\begin{array}{ll} 1 & \Delta ECG\left[n\right]>0\\ 0 & \Delta ECG\left[n\right]=0\\ -1 & \Delta ECG\left[n\right]<0. \end{array}\right. \end{array}$$
(3) First-order forward difference is implemented on the updated ΔECG[*n*], and the value of ΔECG[*n*] is symbolized by −2, 0, and 2. Local maximums in original ECG signal are positions shifted by 1 sample to the right of −2.

**Figure 4:**
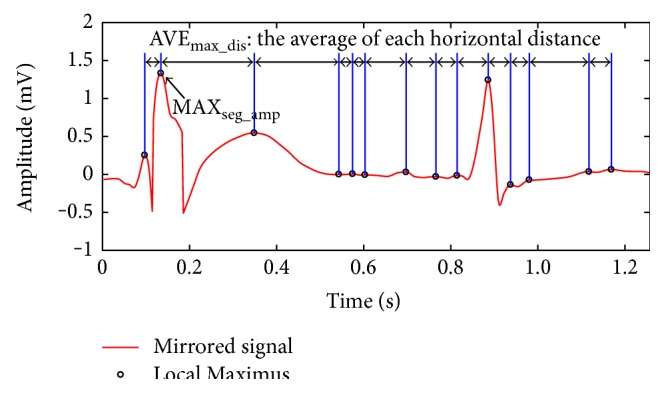
Determination of a_t and ti_t by dimensionless coefficients *K*_amp_ and *K*_time_.

The following threshold procedure depends significantly on the amplitude threshold (a_t) and time interval threshold (ti_t), which are adaptively determined by the location of local maximum instead of adopting fixed thresholds, since fixed thresholds do not copy with large or small amplitude R-peak and slow or fast beat. The selection of the two thresholds is displayed in Figure 4(). During an ECG segment, a_t is selected to be a multiple of the amplitude maximum MAX_seg_amp_; ti_t is selected to be a multiple of the average horizontal distance of each adjacent local maximum AVE_max_dis_. If the positions and amplitudes of these local maximums change, the two thresholds will change correspondingly; hence, a_t and ti_t will adjust adaptively according to the maximum variety.

(3) $$\begin{array}{c} \left\{\begin{array}{c} a\_t=K_{amp}{MAX}_{\mathrm{seg}\_amp}\\ ti\_t=K_{time}{AVE}_{\max\_dis}. \end{array}\right. \end{array}$$

However, the threshold selection is strongly dependent on the noise; *K*_amp_ and *K*_time_ are coefficients designed to correct the noise influence. The detailed selection method for them is discussed in Section 4.3. In a segment, the positions of local maximums are fixed, correspondingly; MAX_seg_amp_ and AVE_max_dis_ are deterministic. Hence, only *K*_amp_ and *K*_time_ need to be decided. The thresholds are automatically updated by the shift of new coming segment. The superiority of automatic threshold substitution embodies in the corresponding adjustment on recognition for small amplitude and slow or fast cardiac beat, as fixed thresholds may fail to detect R-peaks in these cases.

### 2.4. Step 4: Threshold Recognition

**Figure 5:**
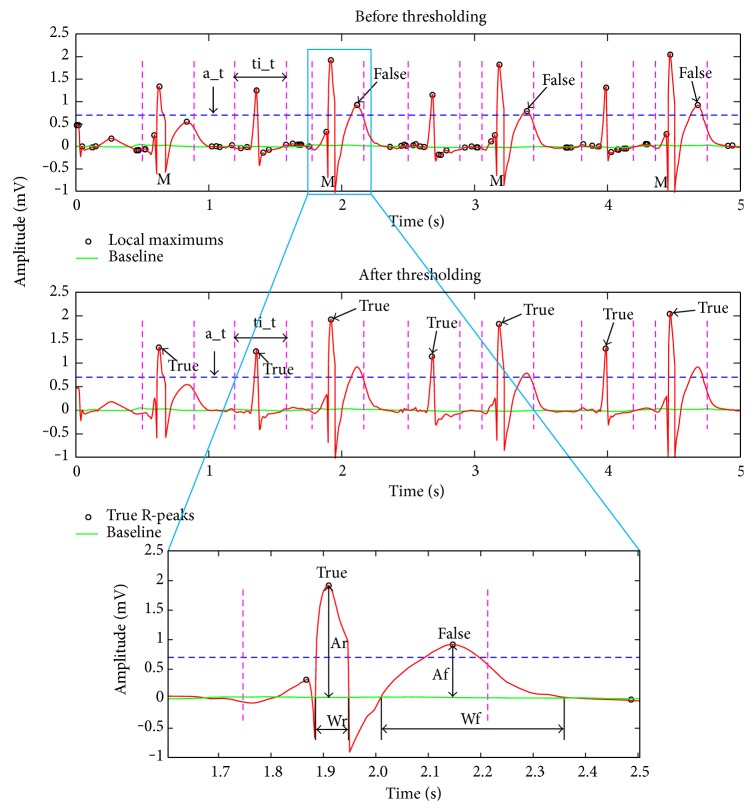
Small amplitudes filtration by a_t and false R-peak exclusion by ti_t.

Actually, most of the local maximums are not true R-peaks, such as burst points caused by high-frequency interference. The difficulty of R-peak detection lies in the recognition of false R-peaks with amplitudes approximate to or even larger than true R-peaks. To this end, a_t is designed to filter the local maximums with small amplitudes. In general, there should be no extra R-peaks between two adjacent R-peaks; otherwise, the extra R-peaks are definitely false. Assisted by this knowledge, ti_t is designed to further remove false R-peaks omitted by a_t. The thresholding algorithm is plotted in Figure 5. The example ECG is from the Record 200 in MITDB with PVC beats. It comprises of large negative R-peaks, and consequently, the signal needs to be mirrored. The marks *M* in Figure 5 signify the mirrored R-peaks, where they should originally be large negative amplitudes. After the amplitude filtration, the time interval threshold algorithm is summarized as follows:

1. *Step A*. A local maximum and its following maximum are chosen as true reference (*Tref*) and comparative reference (*Cref*) respectively, turn to Step B. If there is no *Cref*, *Tref* is considered as a true R-peak and the algorithm ends.
2. *Step B*. The time period (*t*_*p*) between *Tref* and *Cref* is calculated. If *t*_*p*<*ti*_*t*, it indicates that one of the two maximums is a false R-peak, then turn to Step C. Otherwise, *Tref* is considered as a true R-peak, *Cref* replaces *Tref* as true reference, then turn to Step A for next thresholding.
3. *Step C*. Widths along the baseline of *Tref* (*Wr*) and *Cref* (*Wf*) are compared, if *Wr*<*Wf*, *Tref* is considered to be a true R-peak, if *Wr*>*Wf*, *Cref* is treated as a true R-peak [34]. Then turn to Step E. If *Wr*=*Wf*, turn to Step D.
4. *Step D*. Amplitude of *Tref* (*Ar*) and *Cref* (*Af*) is compared, if *Ar*>*Af*, *Tref* is considered to be a true R-peak, otherwise, *Cref* is treated as a true R-peak [34]. Then turn to Step E.
5. *Step E*. The false maximum is replaced by the third local maximum (*Rref*) just behind the two maximums, which is treated as a new *Cref*, and then return to Step B. If there is no *Rref* in the time period, turn to Step A[Fig pseudo1].

## 3. Experiment Designs

### 3.1. Datasets

The MITDB comprises 48 ECG records, and each contains 30-minute ECG signal [35, 36], resulting in a total of 109966 beats that were all used. The ECG records have acceptable quality, sharp and tall P and T waves, negative R waves, small R-peak amplitudes, wider R waves, muscle noise, baseline drift, sudden changes in heartbeat morphology, multiform PVC, long pauses, and irregular heart rhythms [25].

The QTDB contains a total of 105 15-minute ECGs. ECGs in this database were chosen to represent a wide variety of QRS and ST-T morphologies with real-world variability to challenge the detection algorithms [35, 37]. A total of 86995 beats from 82 records were used, and the rest 23 records of sel30-sel52 were excluded since the QRS annotations were not given.

It should be noted that both databases provide two channels of ECG signals. In this study, only the first channel was used for algorithm development and test.

### 3.2. Evaluation Indices

Experimental results are evaluated in terms of sensitivity (SEN), positive predictivity (+P), and accuracy (ACC). The definitions of the indices are expressed in

(4) $$\begin{array}{c} SEN=\frac{TP}{TP+FN}\times 100\%, \end{array}$$

(5) $$\begin{array}{c} +P=\frac{TP}{TP+FP}\times 100\%, \end{array}$$

(6) $$\begin{array}{c} ACC=\frac{TP}{TP+FN+FP}\times 100\%, \end{array}$$

where TP (true positive) is the number of R-peaks correctly recognized, FN (false negative) is the number of R-peaks missed, and FP (false positive) is the number of false R-peaks recognized as true R-peaks. The TP, FN, and FP, verified by the annotations announced in [25], are calculated based on a tolerance window of 50 ms.

Time complexity is also tested, which quantifies the amount of time taken by an algorithm to run as a function of the length of string representing the input. It reflects the increment of time consumption when the input data increase. Time complexity of an algorithm is commonly expressed using *O* notation. If the number of input data *n* multiplies, the time consumption multiplies with an increment of *n* ^*m*^, the algorithm is called to have an *m*-order time complexity symbolized as *O*(*n* ^*m*^). In this study, all the time cost experiments were carried out on a desktop (CPU i7-2600 3.40GHz, 8GB RAM, 64-bit Windows 7 Enterprise) installed with Matlab 2016b.

## 4. Results

First, for both databases, *K*_amp_ and *K*_time_ were initially set as 0.25 and 0.45, respectively. The length of shifting signal was set as 10 s for each thresholding operation. Then, we tested the influences of the parameters *K*_amp_ and *K*_time_.

### 4.1. R-Peak Detection Results

The testing results on MITDB are summarized in Table 1. The results demonstrate a satisfactory performance on the records. The algorithm has a total detection failure of 1229 beats (668 FN beats and 561 FP beats) out of 109966 beats; the average SEN, +P, and ACC are 99.39%, 99.49%, and 98.89% respectively.

The testing results on QTDB are shown in Table 2. The algorithm has a total detection failure of 238 beats (147 FN beats and 91 FP beats); out of 86995 beats, the average SEN, +P, and ACC are 99.83%, 99.90%, and 99.73% respectively. Compared with MITDB, the ECG signals from QTDB have much better waveforms with higher quality; distractors such as motion artifacts, burst noise, large P, and T waves are much less. Consequently, the algorithm achieves a more satisfactory performance over QTDB.

Our algorithm is also compared with several existing methods, including the most widely used Pan-Tompkins method, as shown in Table 3. The comparison indicates that our algorithm achieves comparable high performance.

### 4.2. Time Consumption and Time Complexity

**Figure 6:**
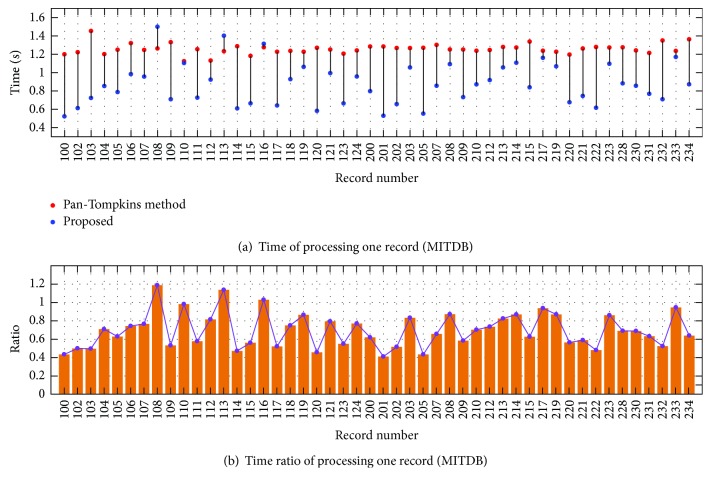
(a) Time consumption of processing the 48 records in MITDB and (b) time consumption ratio of the proposed method over the Pan-Tompkins method.

The time consumption for each record from MITDB is described in Figure 6(a). In general, the Pan-Tompkins method consumes more time than our method for most records. The mean time of this method is 1.256 s to process one record, while our algorithm consumes 0.872 s, achieving about 30.6% of time reduction.  Figure 6(b) shows the time consumption ratio of the proposed method over the Pan-Tompkins method. It is obvious that our method consumes less time for most records except for records 107, 109, 113, and 116, which contain large T waves that cause more frequent thresholding manipulations. In some cases, our algorithm economizes nearly 50% of time than the Pan-Tompkins method.

**Figure 7:**
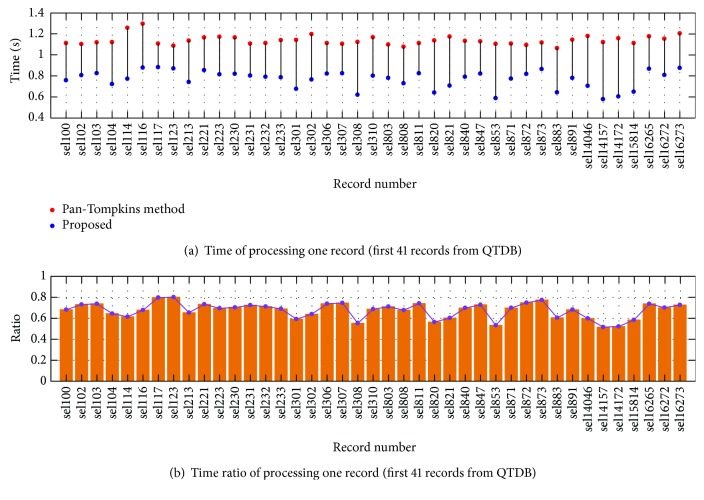
(a) Time consumption of processing the first 41 records in QTDB and (b) time consumption ratio of the proposed method over the Pan-Tompkins method.

**Figure 8:**
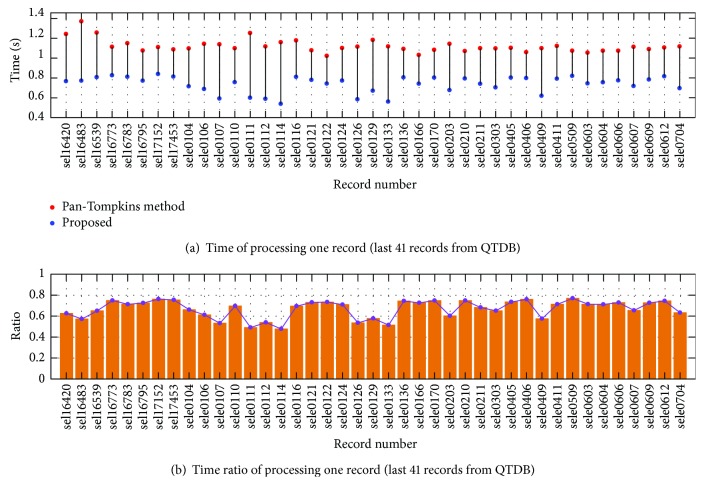
(a) Time consumption of processing the last 41 records in QTDB and (b) time consumption ratio of the proposed method over the Pan-Tompkins method.

The time consumption for each record from QTDB is described in Figure 7(a) and Figure 8(a). It is obvious that our method consumes less time than the Pan-Tompkins method for all the records. The mean time of the Pan-Tompkins method is 1.137 s to process one record, while our algorithm consumes 0.763 s, achieving about 32.9% of time reduction.  Figure 7(b) and Figure 8(b) show the time consumption ratio of the proposed method over the Pan-Tompkins method. It can be seen that all the ratios are less than 1. The outstanding performance can be attributed to the high-quality ECG signals in QTDB.

The time consumption reveals an important characteristic of the two methods. The number of sampling points of each QTDB ECG is 225000, and the number is 650000 of each MITDB ECG. Although the number has increased about two times from QTDB to MITDB, the time consumed increases only 12.5% using our method and 9.2% using the Pan-Tomkins method. It indicates that when data multiplies, the time consumption increases slightly instead of multiplying correspondingly. Both our method and the Pan-Tomkins method are not so sensitive to data increase.

**Figure 9:**
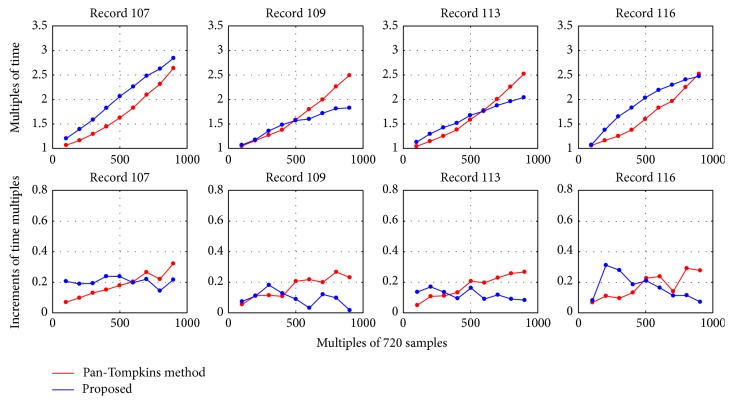
Multiples of time consumption versus multiples of samples.

However, for records 107, 109, 113, and 116 in MITDB, our method consumes the same and even more time than the Pan-Tompkins method. The disadvantage of our method is plotted in Figure 9 versus the Pan-Tompkins method in terms of time complexity. In each subfigure, the abscissa represents the quantitative increment of samples. The basic number of samples is 720; it is multiplied by the number shown in the abscissa. In the top row, the ordinate is the multiple increments of time consumed by processing the multiplied samples shown in the abscissa. In the bottom row, the ordinates are the increment of time multiples calculated from the ordinates in the top row. Intuitively, the four records have high increments of time consumption as the amount of data increases, especially when the data quantity is less than 500 times of the basic number 720. One important reason is that the four records contain numerous large T waves or P waves to be compared by time interval threshold or large negative amplitudes to be estimated for mirroring. Time interval comparison is more frequent for these waveforms, leading to higher time complexity than that of the Pan-Tompkins method. But on the other hand, the curves prove that our algorithm is not so sensitive to data increase. The increments of time multiple fluctuate in small ranges, basically remaining unchanged. The multiples of time consumption have linear relationships with the increments of sample amount.

### 4.3. *K*_amp_ and *K*_time_

Amplitude threshold takes a significant role in truncating burst points along the baseline; time interval threshold is a critical measure to further distinguish false R-peaks. According to (3), the thresholds depend significantly on *K*_amp_ and *K*_time_. To determine optimal coefficients and validate the feasibility of adaptive thresholding, different a_t and ti_t values are tested using the 48 ECG records in MITDB. RR intervals of the records range from 0.54 s to 1.19 s; an average of 0.82 s is adopted to cope with different heart rates. The validation results are summarized in Table 4.

It can be seen that the optimal a_t is generally *K*_amp_ = 0.2 ~ 0.3 times the MAX_seg_amp_, with *K*_amp_ = 0.25 reaching the maximum SEN, +P and ACC as illustrated in Table 1. On one hand, large a_t is beneficial for the algorithm to cut off points located near the baseline, but small true R-peaks may be missed if a_t is too large. As shown in the table, when *K*_amp_ is larger than 0.40, most of the false R-peaks are filtered as well as true ones, resulting in high FN but low FP and correspondingly low SEN but high +P. On the other, smaller a_t generates fewer omitted R-peaks, but this will increase the computational cost in time interval thresholding. The worst situation is that all the potential R-peaks are compared, as revealed in the table when *K*_*amp*_ is smaller than 0.20. In this case, most of the true R-peaks are included in local maximums as well as false ones, resulting in low FN but high FP and correspondingly high SEN but low +P. To coordinate with different heart rates, *K*_amp_ is recommended to be selected from interval [0.2, 0.3] and *K*_time_ from [0.42, 0.48]. After *K*_amp_ and *K*_time_ are determined, a_t and ti_t can be automatically updated by the shift of new coming signal.

## 5. Discussion

The proposed method has two advantages. One is from the time efficiency as indicated in Section 4.2. The main difference between our method and the Pan-Tompkins method is that the latter calculates more measures for R-peak recognition. It includes a search back operation after a complete detection circulation, thus resulting in a high computational complexity. Our method exclusively uses the thresholding method, and it does not require any search back operation. Besides, the amplitude threshold can also contribute to the calculation efficiency since it excludes most distractors and significantly reduces the amount of threshold comparisons.

Another advantage is from that there is no time length limitation for thresholding. As described in Section 2.4, the length of new coming segment is flexible, and the thresholding procedure can operate not only for a single heartbeat but also for a complete ECG record. With adaptive *K*_amp_ and *K*_time_, our method is suitable for different lengths of ECG signal and it requires no prelearning procedure.

From Table 1, we can see that about half of the failed beats (314 FN and 328 FP) are from record 207. This record consists of numerous distorted heartbeats that are extremely difficult to be recognized even by a cardiologist. However, few literatures reported the results on this specific record. It is also unclear if record 207 is excluded in the evaluations to achieve a high score. This record is also the main interference that significantly reduces the detection accuracy of our method.

**Figure 10:**
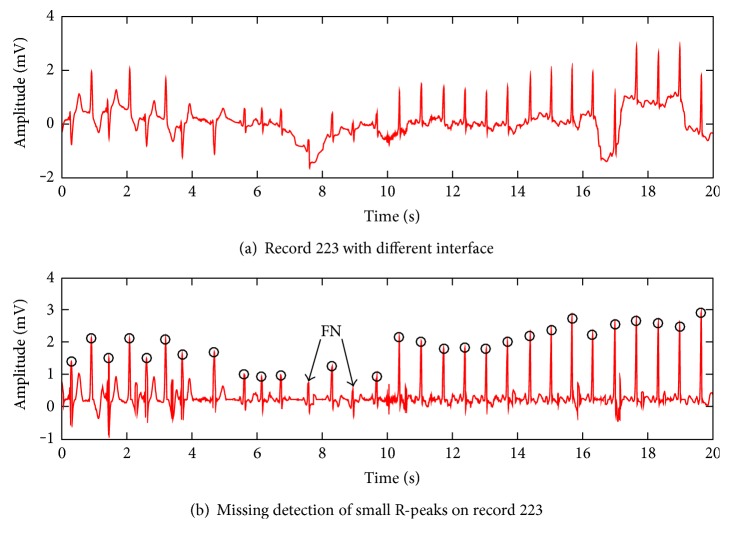
Missing detection due to the small amplitude of R-peaks.

Apart from record 207, there are still some missing and false recognitions on some other records. The main methodological defect of the algorithm is that amplitude threshold may fail to detect small R-peaks mixed in large ones (records 105, 106, 108, and 228). The a_t is selected based on the local maximums; if a segment contains numerous large R-peaks, a_t will be larger than small R-peaks. This weights against the identification for small R-peaks because they are prone to be partitioned below the a_t, as illustrated in Figure 10. The signal contains baseline drift, large T waves, and large negative amplitude R-peaks. Although most of the R-peaks are recognized, there are two FN detections for small R-peaks. If an R-peak is missed, ti_t would probably take the following maximum as the candidate R-peak, which actually is a distractor.

## 6. Conclusions

In this study, an adaptive and time-efficient methodology has been developed for automatic ECG R-peak detection. It is an adaptive method integrating WMRA, signal mirroring, local maximum detection, and amplitude and time interval thresholding. The accuracy performances were tested by using ECG records from MITDB and QTDB. Experimental results indicate that the proposed algorithm achieves average SEN, +P, and ACC of 99.39%, 99.49%, and 98.89% for MITDB, and 99.83%, 99.90%, and 99.73% for QTDB, respectively. In addition, time consumption and time complexity of the algorithm are computed to prove its time efficiency. By processing one ECG record, the average time cost is 0.872 s for MITDB and 0.763 s for QTDB, achieving 30.6% and 32.9%, respectively, of time reduction compared to the Pan-Tompkins method. Experiments on time complexity demonstrate that the proposed method is provided with linear time complexity; both our method and the Pan-Tompkins method are less sensitive to data increase.

## Figures and Tables

**Pseudocode 1 pseudo1:**
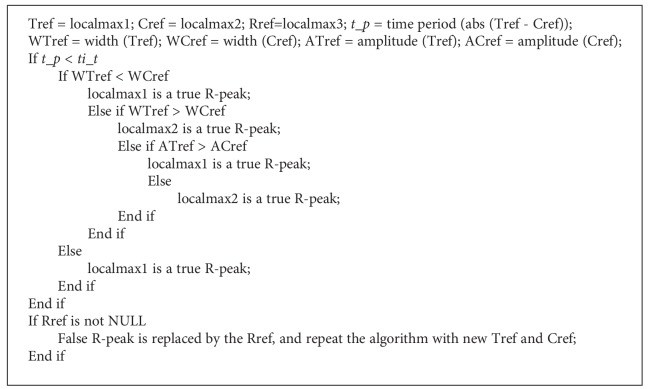
Pseudocode 1: The pseudocode of the threshold procedure.

**Table 1:** Detection results of ECG signals from MITDB.

Record	Total beats	TP	FN	FP	SEN (%)	+P (%)	ACC (%)
100	2273	2273	0	0	100.00	100.00	100.00
101	1865	1864	1	1	99.95	99.95	99.89
102	2187	2187	0	0	100.00	100.00	100.00
103	2084	2084	0	0	100.00	100.00	100.00
104	2229	2222	7	10	99.69	99.55	99.24
105	2572	2528	44	50	98.29	98.06	96.41
106	2027	2004	23	22	98.87	98.91	97.80
107	2137	2121	16	6	99.25	99.72	98.97
108	1763	1739	24	18	98.64	98.98	97.64
109	2532	2532	0	0	100.00	100.00	100.00
111	2124	2117	7	4	99.67	99.81	99.48
112	2539	2539	0	0	100.00	100.00	100.00
113	1795	1795	0	0	100.00	100.00	100.00
114	1879	1872	7	10	99.63	99.47	99.10
115	1953	1953	0	0	100.00	100.00	100.00
116	2412	2393	19	5	99.21	99.79	99.01
117	1535	1534	1	1	99.93	99.93	99.87
118	2278	2277	1	0	99.96	100.00	99.96
119	1987	1987	0	0	100.00	100.00	100.00
121	1863	1860	3	3	99.84	99.84	99.68
122	2476	2476	0	0	100.00	100.00	100.00
123	1518	1518	0	0	100.00	100.00	100.00
124	1619	1617	2	2	99.88	99.88	99.75
200	2601	2593	8	3	99.69	99.88	99.58
201	1963	1962	1	1	99.95	99.95	99.90
202	2136	2123	13	6	99.39	99.72	99.11
203	2980	2953	27	21	99.09	99.29	98.40
205	2656	2640	16	2	99.40	99.92	99.32
207	2332	2018	314	328	86.54	86.02	75.86
208	2955	2932	23	3	99.22	99.90	99.12
209	3005	3005	0	1	100.00	99.97	99.97
210	2650	2629	21	13	99.21	99.51	98.72
212	2748	2748	0	0	100.00	100.00	100.00
213	3251	3245	6	2	99.82	99.94	99.75
214	2262	2253	9	10	99.60	99.56	99.16
215	3363	3360	3	4	99.91	99.88	99.79
217	2208	2193	15	10	99.32	99.55	98.87
219	2154	2154	0	0	100.00	100.00	100.00
220	2048	2048	0	0	100.00	100.00	100.00
221	2427	2417	10	5	99.59	99.79	99.38
222	2483	2480	3	3	99.88	99.88	99.76
223	2605	2585	20	0	99.23	100.00	99.23
228	2053	2032	21	14	98.98	99.32	98.31
230	2256	2256	0	0	100.00	100.00	100.00
231	1571	1571	0	0	100.00	100.00	100.00
232	1780	1778	2	2	99.89	99.89	99.78
233	3079	3078	1	1	99.97	99.97	99.94
234	2753	2753	0	0	100.00	100.00	100.00
Total	**109966**	**109298**	**668**	**561**	**99.39**	**99.49**	**98.89**

**Table 2:** Detection results of ECG signals from QTDB.

Record	Total beats	TP	FN	FP	SEN (%)	+P (%)	ACC (%)
sel100	1134	1134	0	0	100.00	100.00	100.00
sel102	1088	1088	0	0	100.00	100.00	100.00
sel103	1048	1048	0	0	100.00	100.00	100.00
sel104	1109	1109	0	1	100.00	99.91	99.91
sel114	862	858	4	8	99.54	99.08	98.62
sel116	1185	1184	1	1	99.92	99.92	99.83
sel117	766	766	0	0	100.00	100.00	100.00
sel123	756	756	0	0	100.00	100.00	100.00
sel213	1642	1636	6	1	99.63	99.94	99.57
sel221	1247	1240	7	3	99.44	99.76	99.20
sel223	1309	1307	2	2	99.85	99.85	99.69
sel230	1077	1077	0	0	100.00	100.00	100.00
sel231	732	732	0	0	100.00	100.00	100.00
sel232	865	864	1	0	99.88	100.00	99.88
sel233	1533	1507	26	1	98.30	99.93	98.24
sel301	1351	1346	5	1	99.63	99.93	99.56
sel302	1500	1498	2	1	99.87	99.93	99.80
sel306	1040	1040	0	0	100.00	100.00	100.00
sel307	853	853	0	0	100.00	100.00	100.00
sel308	1294	1285	9	5	99.30	99.61	98.92
sel310	2012	1997	15	2	99.25	99.90	99.16
sel803	1026	1026	0	0	100.00	100.00	100.00
sel808	903	902	1	1	99.89	99.89	99.78
sel811	704	704	0	0	100.00	100.00	100.00
sel820	1159	1158	1	0	99.91	100.00	99.91
sel821	1557	1556	1	1	99.94	99.94	99.87
sel840	1180	1179	1	0	99.92	100.00	99.92
sel847	801	801	0	0	100.00	100.00	100.00
sel853	1113	1113	0	0	100.00	100.00	100.00
sel871	917	917	0	0	100.00	100.00	100.00
sel872	990	990	0	0	100.00	100.00	100.00
sel873	859	858	1	1	99.88	99.88	99.77
sel883	892	891	1	2	99.89	99.78	99.66
sel891	1267	1266	1	0	99.92	100.00	99.92
sel14046	1260	1260	0	0	100.00	100.00	100.00
sel14157	1081	1081	0	0	100.00	100.00	100.00
sel14172	663	663	0	0	100.00	100.00	100.00
sel15814	1036	1035	1	0	99.90	100.00	99.90
sel16265	1031	1031	0	0	100.00	100.00	100.00
sel16272	851	851	0	0	100.00	100.00	100.00
sel16273	1112	1111	1	0	99.91	100.00	99.91
sel16420	1063	1063	0	0	100.00	100.00	100.00
sel16483	1087	1087	0	0	100.00	100.00	100.00
sel16539	922	922	0	0	100.00	100.00	100.00
sel16773	1008	1007	1	0	99.90	100.00	99.90
sel16786	925	925	0	0	100.00	100.00	100.00
sel16795	761	761	0	0	100.00	100.00	100.00
sel17152	1628	1628	0	0	100.00	100.00	100.00
sel17453	1047	1047	0	0	100.00	100.00	100.00
sele0104	804	804	0	0	100.00	100.00	100.00
sele0106	896	896	0	0	100.00	100.00	100.00
sele0107	812	806	6	2	99.26	99.75	99.02
sele0110	872	870	2	9	99.77	98.98	98.75
sele0111	907	907	0	0	100.00	100.00	100.00
sele0112	684	675	9	12	98.68	98.25	96.98
sele0114	699	698	1	1	99.86	99.86	99.71
sele0116	558	558	0	0	100.00	100.00	100.00
sele0121	1436	1431	5	0	99.65	100.00	99.65
sele0122	1415	1415	0	0	100.00	100.00	100.00
sele0124	1121	1121	0	0	100.00	100.00	100.00
sele0126	945	945	0	1	100.00	99.89	99.89
sele0129	671	644	27	23	95.98	96.55	92.80
sele0133	840	840	0	0	100.00	100.00	100.00
sele0136	809	809	0	0	100.00	100.00	100.00
sele0166	813	813	0	0	100.00	100.00	100.00
sele0170	897	897	0	2	100.00	99.78	99.78
sele0203	1246	1245	1	1	99.92	99.92	99.84
sele0210	1063	1062	1	0	99.91	100.00	99.91
sele0211	1575	1573	2	4	99.87	99.75	99.62
sele0303	1045	1044	1	0	99.90	100.00	99.90
sele0405	1216	1216	0	1	100.00	99.92	99.92
sele0406	959	959	0	1	100.00	99.90	99.90
sele0409	1737	1737	0	0	100.00	100.00	100.00
sele0411	1202	1202	0	0	100.00	100.00	100.00
sele0509	1028	1028	0	0	100.00	100.00	100.00
sele0603	870	869	1	0	99.89	100.00	99.89
sele0604	1031	1031	0	0	100.00	100.00	100.00
sele0606	1442	1442	0	0	100.00	100.00	100.00
sele0607	1184	1184	0	0	100.00	100.00	100.00
sele0609	1127	1125	2	2	99.82	99.82	99.65
sele0612	751	751	0	0	100.00	100.00	100.00
sele0704	1094	1093	1	1	99.91	99.91	99.82
Total	**86995**	**86848**	**147**	**91**	**99.83**	**99.90**	**99.73**

**Table 3:** Comparison of R-peak detection with other algorithms.

	Dataset	Beats	TP	FN	FP	SEN (%)	+P (%)	ACC (%)
Zidelmal et al. [12]	MITDB	109494	109101	393	193	99.64	99.82	99.47
Pan and Tompkins [15]	MITDB	116137	115860	277	507	99.76	99.56	99.33
Jung and Lee [21]	MITDB	109541	108960	581	579	99.47	99.47	98.94
Chiarugi et al. [38]	MITDB	109494	109288	266	210	99.76	99.81	99.57
Arzeno et al. [39]	MITDB	109517	109099	354	405	99.68	99.63	99.31
Elgendi [40]	MITDB	109985	109738	247	124	99.78	99.87	99.66
Christov [41]	MITDB	110050	109615	240	239	99.74	99.65	99.56
Chouakri et al. [42]	MITDB	110934	109488	1446	3068	98.68	97.24	96.03
Rodríguez et al. [43]	MITDB	44715	42518	879	142	96.28	99.71	97.65
Yeh and Wang [44]	MITDB	116137	115971	166	58	99.86	99.95	99.81
The proposed	**MITDB**	**109966**	**109298**	**668**	**561**	**99.39**	**99.49**	**98.89**
	**QTDB**	**86995**	**86848**	**147**	**91**	**99.83**	**99.90**	**99.73**

**Table 4:** Comparison of different threshold coefficients.

	*K*_time_	*K*_amp_								
		0.10	0.15	0.20	0.25	0.30	0.35	0.40	0.45	0.50
SEN (%)	0.36	98.65	98.93	98.68	97.87	96.45	93.87	90.78	87.54	84.16
+P (%)		80.94	87.29	90.23	92.38	93.69	94.55	96.56	97.89	98.67
ACC (%)		80.06	86.47	89.16	90.56	90.57	89.05	87.94	85.92	83.22
SEN (%)	0.39	96.42	97.59	98.05	97.61	96.31	93.79	90.72	87.51	84.14
+P (%)		85.94	91.24	93.64	94.99	95.58	96.37	98.08	99.04	99.39
ACC (%)		83.28	89.24	91.93	92.83	92.20	90.59	89.14	86.78	83.71
SEN (%)	0.42	95.70	97.20	97.80	97.43	96.18	93.68	90.64	87.44	84.09
+P (%)		89.98	94.56	96.42	97.40	97.79	98.27	98.98	99.38	99.56
ACC (%)		86.48	92.05	94.38	94.96	94.13	92.16	89.80	86.97	83.78
SEN (%)	0.45	94.57	96.47	97.17	*99.39*	95.73	93.36	90.42	87.28	83.94
+P (%)		92.51	96.73	98.28	*99.49*	99.09	99.26	99.42	99.54	99.63
ACC (%)		90.85	96.42	98.54	*98.89*	97.90	95.72	92.94	89.93	86.68
SEN (%)	0.48	93.84	95.87	96.63	96.38	95.26	92.90	89.97	86.86	83.57
+P (%)		93.54	97.20	98.59	99.07	99.26	99.38	99.48	99.56	99.64
ACC (%)		88.13	93.29	95.32	95.52	94.58	92.37	89.55	86.53	83.31
SEN (%)	0.51	93.06	95.28	96.08	95.83	94.69	92.30	89.35	86.24	82.98
+P (%)		94.04	97.45	98.68	99.10	99.28	99.39	99.49	99.56	99.64
ACC (%)		87.87	92.96	94.86	95.01	94.04	91.78	88.94	85.92	82.73
SEN (%)	0.54	92.10	94.59	95.41	95.15	94.02	91.66	88.73	85.66	82.43
+P (%)		94.26	97.63	98.73	99.12	99.30	99.41	99.50	99.57	99.65
ACC (%)		87.21	92.47	94.25	94.36	93.40	91.16	88.34	85.35	82.19
SEN (%)	0.57	90.90	93.54	94.41	94.14	93.03	90.73	87.86	84.85	81.65
+P (%)		94.31	97.68	98.76	99.15	99.31	99.42	99.51	99.57	99.65
ACC (%)		86.17	91.51	93.30	93.38	92.44	90.25	87.48	84.54	81.42
SEN (%)	0.6	89.10	91.87	92.80	92.60	91.58	89.46	86.85	83.99	80.90
+P (%)		94.28	97.67	98.76	99.16	99.32	99.43	99.52	99.58	99.65
ACC (%)		84.53	89.90	91.73	91.88	91.01	89.00	86.48	83.69	80.68
